# Null Hypothesis Testing ≠ Scientific Inference: A Critique of the Shaky Premise at the Heart of the Science and Values Debate, and a Defense of Value‐Neutral Risk Assessment

**DOI:** 10.1111/risa.13284

**Published:** 2019-02-11

**Authors:** Brian H. MacGillivray

**Keywords:** Argument from inductive risk, hypothesis testing, risk assessment, uncertainty analysis, value‐neutral

## Abstract

Many philosophers and statisticians argue that risk assessors are morally obligated to evaluate the probabilities and consequences of methodological error, and to base their decisions of whether to adopt a given parameter value, model, or hypothesis on those considerations. This argument is couched within the rubric of null hypothesis testing, which I suggest is a poor descriptive and normative model for risk assessment. Risk regulation is not primarily concerned with evaluating the probability of data conditional upon the null hypothesis, but rather with measuring risks, estimating the consequences of available courses of action and inaction, formally characterizing uncertainty, and deciding what to do based upon explicit values and decision criteria. In turn, I defend an ideal of value‐neutrality, whereby the core inferential tasks of risk assessment—such as weighing evidence, estimating parameters, and model selection—should be guided by the aim of correspondence to reality. This is not to say that value judgments be damned, but rather that they should be accounted for within a structured approach to decision analysis, rather than embedded within risk assessment in an informal manner.

## 1. SCIENCE, VALUES, AND OBJECTIVITY: IS THERE ANYTHING MORE THAT CAN BE SAID?

Can science be value‐free? Is this even desirable? These simple questions have generated long‐standing debates in philosophy, and carry significant implications for scientific practice and public policy (Douglas, 2009; Hempel, 1965; Lacey, 2005; Rudner, 1953). The arguments vary. Objectivity is the hallmark of science in many classical accounts, where adherence to the rules of logic and principles of inductive reasoning are synonymous with freedom from bias and the pursuit of truth (Quine, 1955). If science is not objective, then its authority is seemingly undermined, as political values or personal interests may be shaping the knowledge that it generates (Reiss & Sprenger, 2017). Or perhaps disinterested scientific reasoning is ill‐suited to respond to the environmental challenges of modernity? Science needs to be normative, on some accounts, for example, how can we characterize risks without an (implicit) judgment that something that we value lies in harm's way (Slovic, 1999)? Indeed maybe the idea of objectivity is a dangerous illusion (Greenland, 2012). Empirical inquiry is after all a different beast from formal logic, and subjective judgments color all stages of the research process (Polya, 1968). Appeals to objectivity may be little more than rhetoric designed to mask underlying value judgments (Havstad & Brown, 2017). Even the fact‐value dichotomy has come under attack, with concepts such as resilience and risk argued to carry both normative and descriptive connotations (Putnam, 2002).

Rather than weigh in on these grand philosophical questions, this article has the more modest aim of critiquing what is known as the “argument from inductive risk” (AfIR), and offering a defense of value‐neutral risk assessment. The latter may seem a rather reactionary and anachronistic stance to take, and is probably at odds with most modern philosophical and social science perspectives of risk (Jasanoff, 1999; Krimsky & Golding, 1992; Wynne, 2001). However, value‐neutrality is likely not a controversial ideal within the risk assessment community, and so a reasonable question to ask is: Why should risk assessors care about what philosophers think of their field? Yet recent years have seen a growing interest in the foundations of risk research from within the discipline (Aven, 2016; Borgonovo, Cappelli, Maccheroni, & Marinacci, 2018; Cox, 2012a; Hansson & Aven, 2014). The question of the role of values in risk assessment is of particular interest because it bears on long‐standing debates about the separation of risk assessment and management, on the objectivity of risk assessment, and on the question of whether risk assessment is a fully‐fledged science (Hansson & Aven, 2014; National Research Council [NRC], 1983, 2009). Nevertheless, one could still ask why risk assessors should care about what *outsiders* think about their discipline. In practical terms, the views of outsiders matter because they play a role in shaping what publics, policymakers, and institutions think about what risk assessors are and should be doing. Risk assessments conducted within government agencies are significantly shaped by laws, regulations, and conventions, and these are developed by committees typically including members drawn from a broad range of disciplines (i.e., not just practicing risk assessors, but also ethicists, legal scholars, economists, subject‐matter experts, etc.) (Albert, 1994; North, 2003; NRC, 1983, 2009). Moreover, risk assessment operates within a broader societal context, which includes a range of interest groups that may invoke arguments from arenas including philosophy and statistical theory. Indeed, the process and practices of risk assessment have been a locus of controversy within legal, political, and administrative institutions, and have often been met with suspicion or distrust from citizen groups, NGOs, industrialists, and scientists (Douglas, 2005; Slovic, 1999). Crucially, this suspicion has often been articulated on grounds relating to values; for example, that risk assessments conceal value judgments, or do not adequately incorporate ethical concerns, or are simply a tool for advancing economic interests at the expense of public and environmental health (Douglas, 2000; Slovic, 1999). And so a clear articulation of the proper role of values in risk assessment could: advance thinking about the foundations of risk analysis; enhance trust in the discipline in public and political spheres; and inform the regulations and conventions that shape risk assessment practice.

Before proceeding with my core argument, I will set out my scope and introduce key concepts and terms. I am *not* arguing that the broader endeavor of risk analysis can or should be free of values. I take it as read that such a position would be ludicrous: questions of which risks to prioritize, how they should be framed, which mitigation options should be considered, how consequences should be evaluated, and which decision criteria to adopt are inextricably value‐laden (Pidgeon, Hood, Jones, Turner, & Gibson, 1992; Shrader‐Frechette, 2012; Slovic, 1999). My focus is restricted to *core scientific inference* within risk assessment, namely, the analysis, synthesis, and interpretation of evidence. I take the view that risk assessments are primarily concerned with making and communicating informative, good predictions. By *informative*, I mean that they offer outputs that are relevant to some real‐world decision problem. By *good predictions*, I mean that they seek to produce reliable statements about the world, in the sense that they aim to correspond with an as yet unobserved reality. These statements relate to *potentially* observable quantities or events, although they may not be testable in practice (Goerlandt & Reniers, 2018; Goldstein, 2011; Popper, 2005). I consider that (1) risk can be defined in terms of a triplet of scenarios, consequences, and probabilities, and that (2) any risk assessment is conditional upon a knowledge base whose uncertainties should be explicitly accounted for (Aven, 2013; Kaplan & Garrick, 1981). I use the term *regulatory science* as shorthand for the range of scientific and technical analyses conducted with the aim of informing public policy. I will talk a great deal of hypothesis testing, as this is the conceptual framework within which the AfIR is both defended and critiqued. There are of course multiple approaches to hypothesis testing, most notably Fisher, Neyman–Pearson, and the amalgamation known as null hypothesis significance testing (NHST) (for an overview, see Barnett, 1999; Lehmann, 1993). I will (somewhat loosely) refer to the AfIR as an extension of the Neyman–Pearson paradigm as it is based on the question of whether one should treat a hypothesis as true, rather than whether one should (provisionally) *believe* it to be so (Fisher's approach).^1^

The article proceeds as follows. I begin by explicating the concept lying at the heart of the science and values debate—underdetermination—before focusing on the influential AfIR. In its simplest form, the argument states that scientists are morally obligated to evaluate the probabilities and consequences of incorrectly accepting or rejecting the hypothesis under examination, and to base (in part) their decision of whether to accept a given hypothesis on those considerations (e.g., via altering significance thresholds) (Douglas, 2000, 2009; Neyman, 1957; Rudner, 1953; Steel, 2010).^2^ I then briefly consider prominent rebuttals. The core of the article argues that the AfIR is based on several untenable (implicit) assumptions about the aims and practices of regulatory science. These include the belief that the probabilities and consequences of methodological errors can be estimated and accounted for informally; an overly restrictive framing of policy options; and an at best marginal role afforded to formal decision analysis. I argue that these assumptions stem from conceiving of risk assessment within the rubric of null hypothesis testing. But risk regulation is not primarily concerned with evaluating the probability of data conditional upon the null hypothesis, but rather with measuring risks, estimating the consequences of available courses of action, formally characterizing uncertainty, and deciding what to do based upon explicit values and decision criteria. Doing so in a rigorous and transparent manner requires a value‐neutral approach to risk assessment.

## 2. UNDERDETERMINATION, THE AfIR, AND PROMINENT REBUTTALS

At the heart of the science and values debate lies the idea that the evidence available to us at any given time is insufficient to fix the beliefs that we should hold in response to it, for example, when evaluating a theory, model, or hypothesis (Stanford, 2017). In a sense, this problem of underdetermination reflects the truism that empirical inquiry cannot proceed by deduction alone. Hypotheses only have empirical implications when conjoined with auxiliary hypothesis or background beliefs (e.g., about measurement techniques), and so a failed prediction does not determine which of our beliefs should be updated or abandoned (Duhem, 1991; Laudan, 1990). Some scholars take this to mean that there is a “gap” between evidence and the beliefs that we should form in response to it, and that this gap might as well be filled by “values.” However, values come in many different stripes, some of which are unthreatening to classical notions of objectivity. Epistemic values—those that promote the acquisition of true beliefs (Goldman, 1999)—include notions such as simplicity, testability, and internal consistency. Defenders of the value‐free ideal argue that these principles of inductive reasoning, together with empirical evidence, are sufficient to fix beliefs relating to hypotheses without any need for intrusion by “nonepistemic values” (normative values, such as social or ethical ones) (Norton, 2008). However, there is no consensus on the relative importance of epistemic values, nor on how they should be interpreted, nor indeed on what values can be properly considered epistemic (Douglas, 2013; Kelly, 2011; Kuhn, 1977). Yet this merely shows that there is an unavoidable element of judgment in the application of epistemic values, rather than necessarily undermining the standard account of objectivity. To do the latter, one needs to show that normative judgments, such as social or political values, (should) play a role in scientific reasoning. This brings us to the AfIR.

In brief, the AfIR holds that (1) given that the evaluation of a hypothesis is unsettled by logical or evidential considerations, then (2) there is a nontrivial probability of forming erroneous beliefs in relation to it, and by extension (3) scientists have a moral obligation to take the costs of errors (false positives and false negatives) into account in hypothesis evaluation (Douglas, 2000, 2009; Neyman, 1957; Rudner, 1953; Steel, 2010). On this account, social values play a role in handling uncertainty rather than as reasons for belief formation. That is, they determine the evidentiary thresholds that must be met for a hypothesis to be treated as true, based on the consequences of getting it wrong. There are two types of inductive risks—wrongly rejecting a true hypothesis, and wrongly accepting a false hypothesis—and social values properly determine acceptable risks of error in a particular context, with different contexts legitimately calling for a different balance between the two types of error. While Rudner (1953) forwarded this argument in relation to both pure and applied science, most modern versions of it are restricted to science that is developed for the purpose of informing public policy (i.e., “regulatory science”) (Douglas, 2000, 2009; Steel, 2010).

In a sense the AfIR is a restatement of Neyman and Pearson's view that the consequences of error should play a role in evaluating hypotheses (Neyman, 1957; Pearson, 1955). Under this viewpoint, the crucial point is not whether a hypothesis is true, but rather whether one should treat it as though it were true. In other words, hypothesis testing is an act of choice rather than pure inference (Neyman, 1957). As such, consequences and their associated utilities play a crucial role in this account. However, proponents of the AfIR have had little to say on *how* nonepistemic values should be used to set inferential thresholds, seemingly advocating an informal approach (Kaivanto & Steel, 2017).

Jeffrey's (1956) classic rebuttal to AfIR is that scientists should neither accept nor reject hypotheses, but attach probabilities to them, sidestepping judgments about whether claims are certain enough to warrant treating them as though they were true. Standard counter‐responses to Jeffrey include that the (inevitable) existence of second‐order uncertainty—uncertainty about the probability judgments—lets the inductive risk argument return through the back door (Douglas, 2009). This is unpersuasive, at least from a “probabalist” perspective, wherein second‐order uncertainty simply collapses into first‐order uncertainty (i.e., “any set of base and higher level probabilities can be collapsed into a single set of base level probabilities” [Lehner, Laskey, & Dubois, 1996]). Of course, it is true that risk assessors will have more “confidence” in some probability estimates than in others. However, measures of this confidence can be conveyed to decisionmakers, for example, through assertions of the degree to which assessors expect to update their probabilities in the face of new data (Lehner et al., 1996). This is now standard practice in the IPCC's climate change assessments (Mastrandrea et al., 2011). Another counter‐response to Jeffrey is that decisionmakers are notoriously uncomfortable with uncertainty. This implies that were scientists to simply report the relation between evidence and hypotheses to decisionmakers—perhaps in the form of confidence intervals or *p*‐values—they would be sowing confusion (Elliott, 2011). Yet risk communication, however challenging, is arguably a shared responsibility. Risk assessors achieve little if they provide incomprehensible (to decisionmakers) characterizations of uncertainty, but decisionmakers share a responsibility to grapple with uncertainty in appropriate forms.^3^ The final counter‐response to Jeffrey's argument is that there are numerous methodological choices prior to the appraisal of a hypothesis—for example, model selection, interpreting data, choosing parameter values, weighing evidence, and so on (Douglas, 2000). Unlike with hypothesis appraisal, these decisions cannot as a practical matter be left unmade, and so the AfIR slips back in (Douglas, 2009). A rebuttal to this is that rather than relying upon fixed choices, scientists can use a plurality of models, perform sensitivity analysis to reflect parameter uncertainty, and feed this into a formal decision analysis that would explicitly account for values (e.g., utilities) (Betz, 2013; Frank, 2017; Parker, 2010). I find this argument persuasive and will return to it later.

A second rebuttal to AfIR focuses on its technocratic leanings (Betz, 2013). On what moral or political authority are scientists warranted to make judgments about the relative desirability of certain social consequences? Why should scientists’ normative judgments matter, rather than those of the public, stakeholders, or elected representatives? What grounds do we have to believe that they hold expertise in moral reasoning? And should we not be wary of placing such authority in the hands of a group that is distinctly unrepresentative of broader society? Indeed, the importance of distinguishing between risk assessment and risk management has long been emphasized by the risk analysis community (NRC, 1983, 2009). However, is the philosopher king the logical conclusion of the AfIR? Not necessarily, as many of its proponents have written at length on various methods of eliciting or co‐producing value judgments, from citizen juries to participatory modeling exercises (e.g., Douglas, 2009). Nevertheless, in practice these kind of processes tend to be geared more toward questions of framing—for example, what sort of questions are posed, what kind of consequences matter, and what moral calculus should be adopted in decision making—and eliciting explicitly normative values (e.g., in expressed preference surveys, deliberations over whether equity weights should be adopted in health technology appraisal). They have not focused on engaging publics within the core inferential tasks of risk assessment.

A third rebuttal is that AfIR overstates the degrees of freedom available to the typical risk assessor. Many regulatory domains are characterized by legal rules or conventions setting out methodological choices to be adopted under conditions of uncertainty (MacGillivray, 2014, 2017; NRC, 1983, 2009). These include guidance on the alpha level to select in hypothesis testing, hierarchies that set out how different lines of evidence should be weighed, the preferred model to be adopted in dose‐response assessment, rules for aggregating data, and guidelines on what constitutes valid evidence versus junk science. These heuristics serve to constrain interpretive possibilities, conferring a degree of stability, consistency, and transparency to a process that might otherwise be (seen as) highly sensitive to the choices of individual analysts and thus open to legal attack (Albert, 1994). There are, of course, substantive critiques of rule‐bound inference, focusing on the idea that the *uncritical* adherence to methodological conventions is an impoverished version of objectivity, and one that is at variance with the ideal of truth‐seeking. (Feyerabend, 1993; Gelman & Hennig, 2017; Greenland, 2017a, 2017b). Nevertheless, the point is that analytical discretion cannot simply be assumed to exist within regulatory science, suggesting that the discussions of “moral obligations” that are central to AfIR rather miss the point. Even in the absence of (binding) methodological guidelines, conventions may emerge autonomously and wield significant normative force (Franklin, 2016; Saltelli et al., 2008; Thompson, Frigg, & Helgeson, 2016). Nevertheless, this rebuttal only suggests that analytical degrees of freedom are more limited than supposed by proponents of the AfIR, rather than nonexistent. Moreover, the rebuttal at best pushes the AfIR to another level: that of the institutions responsible for establishing these default inference rules and conventions, rather than the analysts who apply them. In other words, the moral obligation for considering the costs and benefits of methodological error would lie with risk regulation institutions. This would be a more sensible argument—given that institutions (rather than individual analysts) in principle have the time, expertise, authority, and resources to evaluate the costs and benefits of methodological errors in an explicit and formal manner—and more or less describes the current state of affairs in many jurisdictions. However, problems arise when the assumptions and scope conditions underlying such conventions are not clearly stated, as well as when they are not well supported by empirical or theoretical evidence (MacGillivray, 2014, 2017). A particular example is that the convention of NHST has crept into aspects of regulatory practice where its underlying assumptions are questionable (i.e., no uncontrolled confounders and zero measurement error) (MacGillivray, 2014, 2017).

Having overviewed the terrain of the debate on science, values, and objectivity, below I present my own critique of the AfIR, and defend a value‐*neutral* approach to risk assessment.

## 3. NULL HYPOTHESIS TESTING IS A POOR DESCRIPTIVE AND NORMATIVE MODEL FOR RISK ASSESSMENT

### 3.1. Risk Assessment Is Primarily Concerned with Estimation, Not Hypothesis Testing

[N]o analysis of what constitutes the method of science would be satisfactory unless it comprised some assertion to the effect that the scientist as scientist accepts or rejects hypotheses. (Rudner, 1953)

On the Churchman‐Braithwaite‐Rudner view it is the task of the scientist as such to accept and reject hypotheses in such a way as to maximise the expectation of good for, say, a community for which he is acting. (Jeffrey, 1956)

Proponents (and critics) of the AfIR typically frame regulatory science as being focused on (null) hypothesis testing. Some of the examples they discuss include: Is a chemical carcinogenic or not (Brown, 2013)? Does a specific level of exposure to a chemical have a toxic effect (Steel, 2010)? Is a drug currently on the market safe (Biddle, 2013)? Is a toxic contaminant present in a drug in a lethal quantity (Rudner, 1953)? Is a vaccine stock free from the active polio virus (Jeffrey, 1956)? While this model of regulatory science—in which things are either safe or unsafe, and unsafe things should be regulated—was perhaps a reasonable one in Rudner's time, it is now broadly untenable. The most famous example of this categorical or absolutist approach to risk regulation is probably the U.S. Delaney Clause:

No additive shall be deemed to be safe if it is found to induce cancer when ingested by man or animal, or if it is found, after tests which are appropriate for the evaluation of the safety of food additives, to induce cancer in man or animals.

This clause soon became untenable as advances in toxicology and analytical chemistry revealed that there were many more carcinogens present in foodstuffs than initially expected and that there were marked differences in their potencies (Majone, 2010). As a result, absolutist rules now play a more limited role in risk regulation, replaced by a broad acceptance that decision making should be based on *levels* of risk and associated cost‐benefit considerations (Graham & Wiener, 1995). This is not to say that hypotheses play no role in risk assessment (MacGillivray, 2017; Spiegelhalter, Abrams, & Myles, 2004; Suter, 1996). For instance, does a spike in hospital mortality rates indicate an underperforming institution (e.g., substandard surgical practices or conditions), or is it random variation? Can a change in weather patterns (e.g., altered frequency or strength of North Atlantic storms) be attributed to anthropogenic causes, or is it within the bounds of natural system behavior? In the context of pharmacovigilance, has a drug‐event pair been reported disproportionately? Variants of null hypothesis testing are widely used to structure these kinds of inferences, particularly where data are generated by randomized experiments (in theory ruling out systematic error) and there is limited prior information. Such practices have been subject to the standard critiques of: using arbitrary thresholds to discriminate between signal and noise; adopting strong assumptions of no uncontrolled confounders and zero measurement error (particularly implausible when observational data are used); only indirectly considering statistical aspects that are logically informative for causal inference (e.g., effect sizes in clinical trials); and ignoring priors and utilities (Gigerenzer & Marewski, 2015; Spiegelhalter et al., 2004; Suter, 1996). A long‐established but often overlooked principle is that *p*‐values are *not* measures of the truth or probability of the null hypothesis, but rather measures of the probability of the data conditional upon the truth of the null hypothesis (and auxiliary assumptions) (Greenland et al., 2016). This measure will not always have direct relevance for policy making. As such, null hypothesis testing is less often used as a strict decision procedure within risk assessment, but rather as one line of evidence among many that contribute to causal inference. Causal inference in practice is typically guided by domain‐specific criteria—such as Koch's postulates (Doll, 2002) or Hill's (1965) criteria—rather than the ritualistic application of significance levels (although abuses remain [MacGillivray, 2017; Suter, 1996], perhaps because NHST better fits a desire for absolute safety). Moreover, establishing causation is often the starting rather than end point of risk assessment, where the fundamental question of interest is what is the *level* of risk posed by a process, product, or activity, rather than simply whether a causal association has been demonstrated.

### 3.2. Hypothesis Testing Frameworks Neglect Bias and Focus on Random Error; The AfIR Inherits These Weaknesses

Recall that risk assessment involves numerous unforced methodological choices to which the AfIR putatively applies (Douglas, 2000). The implications are that scientists are morally obliged (within legal constraints) to (1) estimate probabilities of over‐ or underestimating a parameter value; (2) estimate the consequences of those errors; (3) evaluate those consequences (in a normative sense); and (4) select an optimal parameter value in light of some (unspecified) decision criteria. And all of this should be repeated for uncertain model choices, questions about how to characterize ambiguous data, disputes over which extrapolation method to use, and so on, seemingly without the aid of formal uncertainty analysis (see also Kaivanto & Steel, 2017).^4^ What are the problems with this? Null hypothesis testing frameworks—or *p*‐values more accurately—provide a measure of the probability of obtaining data at least as extreme as that observed, given that the null hypothesis is true (e.g., that a parameter lies within a certain range), and conditional on assumptions of no uncontrolled confounders and zero measurement error (Greenland et al., 2016). The only uncertainty that they formally express is that of random error, leaving uncertainty surrounding the auxiliary assumptions to be handled informally. This is hard to justify when risk assessments frequently rely on noisy data—often from proxy variables rather than the attributes of direct interest—obtained from nonexperimental settings, where random error will typically be a second‐order problem compared to measurement error and bias (Greenland, 2005; Lash, Fox, Cooney, Lu, & Forshee, 2016). Formal approaches to uncertainty analysis would help (Betz, 2013; Frank, 2017; Parker, 2010), and indeed are widely applied in (best) practice.

For example, parametric uncertainty is widely handled through sensitivity analysis. Conventionally this is done through varying one parameter or input value at a time over an arbitrarily limited space, but this is strictly speaking inadvisable for correlated sources of error (Saltelli et al., 2008). “Global sensitivity analysis” (GSA) is a promising method for covering a fuller (though still incomplete) space of parameter uncertainty and considering correlated sources of errors. Moreover, it does not require the specification of (often arbitrarily chosen) probability distributions. GSA produces a set of outcomes believed to be plausible (or at least not implausible) conditional on underlying model structure.^5^ However, it may be computationally demanding to apply (Saltelli et al., 2008). The use of emulators may be a reasonable compromise (Coutts & Yokomizo, 2014), particularly in situations where the underlying model is well‐calibrated against observations, and where further observations can be used to benchmark the performance of the emulator itself.^6^ Box's (1979) aphorism that all models are wrong (but some useful) implies that characterizations of uncertainty that are conditional on the truth of a model are insufficient, particularly when dealing with tasks of extrapolation or out‐of‐sample prediction (Greenland, 2005). And so logic trees are sometimes used to convey how the distribution of risk estimates is conditional on unforced methodological choices at multiple stages throughout risk assessment. Directed acyclic graphs together with structural equation modeling can be used to explore the sensitivity of risk estimates to violations of standard assumptions of no measurement error and no uncontrolled confounding (Pearl, 2009; VanderWeele & Ding, 2017). Alternatively, ensemble methods may be used to provide a (lower‐bound) characterization of model uncertainty, most famously in climate science (Knutti, 2010; Morgan & Henrion, 1990). Structured approaches to eliciting and aggregating expert judgments—for example, probability distributions of uncertain parameters that correspond to real‐world variables,^7^ or of the weights that should be applied to alternative models in ensemble forecasting—can provide rigor and transparency to a process that may otherwise be hostage to cognitive biases and groupthink (Aspinall, 2010; Clemen & Winkler, 1999; Morgan, 2014). A particularly challenging type of model uncertainty stems from the omission of physical processes that are thought to be significant, yet that are insufficiently understood to allow for formalization. The method of probabilistic inversion—used to combine expert judgments with the outputs from physical models—offers a coherent, reproducible basis for correcting for the biases stemming from such omissions (e.g., through adjusting mean estimates or widening distributions) (Oppenheimer, Little, & Cooke, 2016). More recent attention has been placed on the conditionality of analysis outputs to arbitrary choices in data processing, given that converting raw observations into data sets amenable to formal analysis often involves many unforced choices (Gelman & Loken, 2014). The robustness (or fragility) of analysis outputs can be clarified by reporting the results for all reasonable data sets, rather than a single data set that arises from unforced data‐processing choices (Steegen, Tuerlinckx, Gelman, & Vanpaemel, 2016). These methods require a degree of sophistication to apply. Good practice, after all, requires hard work.

The general point is that there are formal methods for uncertainty analysis, which to a large extent remove the obligation of the analyst to estimate, through untutored introspection, the probabilities, and consequences of methodological error. However, the complexity of the problems that most risk assessments deal with means that we are rarely in a situation where all sources of uncertainty can be fully, formally characterized. Extensive formal uncertainty analysis is resource intensive, and the length required to conduct quantitative risk assessments is already a major concern in some domains. As such, the resources expended on formal uncertainty analyses should be commensurate with the scale of the problem under analysis, and the value of information that such analyses might provide (NRC, 2011).

Many proponents of the AfIR appear motivated by the view that decisionmakers desire precise, definitive analysis outputs and that transparent, rigorous characterizations of uncertainty will only sow confusion. Whether this view is correct is disputed within the risk assessment community. One founder of the domain of carcinogen risk assessment argued that risk managers “do not like uncertainty because it makes it difficult to formulate and defend regulatory action” (Albert, 1994; for similar arguments, see Goldstein, 2011). Others have found decisionmakers to be capable of interpreting probabilistic statements, and surprisingly receptive to risk assessments that clarify underlying assumptions and uncertainties (Stirling, 2010). My own belief is that risk assessments that do not explicitly acknowledge uncertainties and potential biases are misleading, regardless of what decisionmakers’ preferences may be (see also Finkel, 1995; Jasanoff, 1989; Morgan, 2018).

### 3.3 The NHST Framework Neglects Substantive Features of Regulatory Decision Making; Formal Decision Analysis Is a Superior Model

Fundamentally, the AfIR is concerned with whether one should treat a hypothesis (or risk estimate) as though it were true, rather than whether one should believe it to be so, putting it squarely within the realm of decision theory. However, the AfIR is typically discussed within the rubric of null hypothesis testing, wherein questions of consequences, utilities, and decision criteria are addressed informally if at all, and the decision in question is whether to accept or reject the hypothesis under examination. Why does this matter? Proponents of AfIR as a consequence tend to adopt a model of regulatory decision making wherein risk assessors are responsible for testing hypotheses (effect vs. no effect) or estimating risk levels, the outputs of which are sufficient for decision making, and where choice is reduced to the question of whether to regulate or not. Is this idealized picture obscuring significant details?

To begin with, are there contexts in which estimates of the level of risk are *sufficient* for decision making? In some domains and jurisdictions quantitative thresholds^8^ are indeed used to distinguish between negligible risks and those that require mitigation, based either on theoretical grounds (e.g., threshold models of risk, or “tipping points”), pragmatic concerns (e.g., analytical detection limits), or arbitrary conventions (e.g., an upper‐bound lifetime incremental cancer risk of 10^−6^ has been used as a measure of unacceptable risk by many U.S. regulatory agencies) (MacGillivray, 2017; Rodricks, Brett, & Wrenn, 1987). Although *de minimis* thresholds can be justified on the grounds that “the law does not concern itself with trifles”(Peterson, 2002), *de manifestis* thresholds are often on shakier ground, given their typically arbitrary foundations and explicit neglect of the risk management options on the table as well as their associated cost‐benefit and equity considerations.

Indeed, even where such thresholds are dictated by law, agencies often circumvent them via creative statutory interpretations, preferring to make their decisions on the basis of (officially forbidden) factors such as costs, benefits, and equity considerations (Coglianese & Marchant, 2004). This is to say that regulators are rarely simply interested in estimates of risk, but rather in the outcomes expected to follow from their (potential) actions, and of how they might be valued. These are counterfactual questions with an explicit component of valuation, rather than questions of pure inference, and so are surely properly handled within the apparatus of decision analysis, wherein regulatory options are specified, uncertainty (parameter and model) characterized, consequences calculated and valued (e.g., by assigning utilities), and options ranked with respect to some agreed upon criteria (see also Definetti, 1951; Jeffrey, 1956).

Moreover, a further weakness of the AfIR is that its proponents typically portray false positives (overregulation) as merely imposing economic burdens on the regulated industry, while portraying false negatives (underregulation) as incurring a range of social, environmental, and health impacts (Biddle, 2013; Brown, 2013; Douglas, 2000; Frank, 2017; Hicks, 2018).^9^ The implication is that in the context of environmental and public health protection, false negatives will generally prove more costly than false positives, and that significance levels should be set accordingly. While these assumptions are not a necessary component of the AfIR, they are pervasive and worth examining.

To begin with, the long‐standing (and controversial) argument that wealth = health (e.g., Lutter, Morrall, & Viscusi, 1999) poses a challenge to their assumptions. The rough idea is that imposing (unwarranted) burdens on industry lowers productivity and by extension the income levels of the public. Given that wealthier people tend to live longer and healthier lives, it follows that false positives may impose welfare burdens beyond simply economic harm to industry. More simply, many risk reduction measures incur significant direct costs to the state, whether structural measures designed to protect coastal areas from flooding, pharmacovigilance systems designed to monitor for adverse drug outcomes, or the infrastructure of buoys, cables, alarms, simulation models, and shelters that make up modern tsunami warning systems. In a world of constrained resources, (unnecessary) expenditures divert public funds that could have been used to advance social welfare through other means (Tengs et al., 1995). Yet the problem of tradeoff neglect is broader than this. A defining feature of modernity is that we are engaged in transforming risks rather than solving them, in managing tradeoffs between risks, in substituting one set of risks with another, and in shifting harms from one jurisdiction to the next (Busby, Alcock, & MacGillivray, 2012; Graham & Wiener, 1995; MacGillivray, Alcock, & Busby, 2011; Sunstein, 1990; Viscusi, 1996).^10^

None of the above is intended to suggest that overregulation is a larger concern than underregulation.^11^ Instead, the foregoing examples are intended to support a long‐standing criticism of the AfIR, dating back to Jeffrey (1956) and DeFinetti (1951), that we need to know the specific actions being considered before we can meaningfully estimate the consequences of error. For example, flood risk can be handled either by population resettlement, watershed management practices, structural measures such as levees and dams, improved emergency planning and evacuation procedures, or some combination therein. The consequences of methodological error—for example, of overestimating risk levels—will differ depending on which intervention is under consideration (Definetti, 1951; Finkel, 2011; Jeffrey, 1956). The AfIR seems to imply that in such contexts, analysts should provide multiple estimates, conditional on each potential intervention, that take the variable consequences of error into account, including tradeoffs and side‐effects. This is surely cognitively intractable.

A separate challenge is that risk assessment outputs can take on a life of their own, traveling across boundaries and scales to be applied in decision contexts far removed from those originally intended. On this account, regulatory science is not so different from pure science, in that the domains of potential application are heterogeneous (with different loss functions) and cannot reasonably be foreseen in advance, presenting further difficulties for the AfIR. Moreover, even within a given context, there will be multiple audiences with diverse interests and values. Surely the more reasonable alternative is to produce value‐neutral risk assessments and to incorporate societal values, loss functions, and equity considerations within formal decision analysis frameworks where possible.

A natural counterargument to the above is that formal decision‐theoretic methods are only applicable to a relatively limited subset of problem types, where states of the world, available choices, and their associated consequences and probabilities are known to the decisionmaker (Savage's [1972] “small worlds”). In “large worlds,” characterized by uncertainty relating to these problem dimensions, Gigerenzer and colleagues (e.g., Gigerenzer & Marewski, 2015) have claimed that Savage viewed the application of the full Bayesian apparatus as “utterly ridiculous.”^12^ This, however, misreads or at least overstates Savage's point. Savage argued that in order to apply Bayesian methods to large worlds, we need to make various simplifying assumptions so that they can be analyzed *as if* they were small worlds. This involves, for example, describing states of the world and consequences stemming from potential actions at some fixed and idealized level of detail (Shafer, 1986). Without doing so, the application of Bayesian methods would be “utterly ridiculous” as the problem structure would be ill‐defined and the task intractable. The basic point is that while it is true that probability and decision theory can never solve problems of actual practice, they can in fact solve idealizations of those problems. And so the application of these approaches is valuable to the extent that those idealizations are good ones and can be communicated to interested parties (Jaynes, 2003; Savage, 1972). Using formal decision‐theoretic apparatus to identify a single “optimal” policy in situations of deep uncertainty—where boundary and initial conditions are poorly understood; parameter values weakly constrained by theory or empirics; and model structures contain significant omissions—is an act of faith rather than of science (Freedman, 1997). In such situations, inexact methods of problem solving may be more defensible (Jaynes, 2003). Precautionary approaches—for example, heuristic decision rules based on feasibility standards or worst‐case scenarios (Kysar, 2010; MacGillivray, 2017)—may prove useful where the costs of underregulating are likely to dwarf those of overregulating (e.g., tsunami early warning systems), and minimax or low‐regret principles can enhance their rigor and transparency (Heal & Millner, 2014). Another alternative is robust decision making, which involves selecting policies that perform well across a wide range of plausible outcomes. These frameworks have appealing properties in conditions of deep uncertainty, and do not depend on characterizing uncertainty via probability distributions (Cox, 2012b; Heal & Millner, 2014; Lempert & Collins, 2007). A final alternative is sequential strategies, which build flexibility into decision making through staged implementation of mitigation efforts, leaving space for adaptation to changing conditions (Simpson et al., 2016). The general point is that risk assessments are typically not a direct input to the decisionmaker, but rather fed into a broader analysis framework wherein decisionmakers’ (or societal) preferences are explicitly incorporated, for example, through assigning utilities, measures of risk aversion and equity, and so forth, or informally incorporated via structured decision processes. The corollary of this is that risk assessors are morally obliged to be value‐neutral in their methodological choices and to make clear what those choices are, rather than to impose their own preferences.

## 4. CONCLUSIONS

The AfIR states that risk assessors are morally obligated to evaluate the probabilities and consequences of methodological error, and to base decisions of whether to *act* as though a given parameter, model, or hypothesis is true on those considerations (e.g., via altering significance thresholds). Proponents of this argument express and defend their claims within the rubric of null hypothesis testing, which I have argued to be a poor descriptive and normative model for risk assessment. It is a poor model because it only indirectly considers effect sizes; rests on typically implausible assumptions of no measurement error and no uncontrolled confounding; answers a question that is often of little substantive interest to policy making; neglects utilities; and restricts the choice‐set to whether one should treat the null hypothesis as true or false. I also claimed that the AfIR places unreasonable cognitive demands on risk assessors. Risk assessment involves multiple complex inferences, which may combine in nonintuitive ways, with multidimensional impacts and varied tradeoffs, and so the idea that analysts can reliably foresee the implications of methodological errors seems questionable. The argument also rather misses the point because the application of formal uncertainty and decision analysis in regulatory contexts already systematizes this practice, in a way that (ideally) rigorously and transparently acknowledges uncertainty, and that ranks regulatory options based upon agreed upon decision criteria and explicit evaluations of consequences. My general normative argument has been that risk assessment should aspire toward value‐neutrality. This means that the core inferential tasks of risk assessment—such as weighting data, estimating parameters, and model selection (or combination)—should be guided by the aim of correspondence to reality. Epistemic pluralism—in the sense of openly accounting for the range of plausible methods, data, parameter values, and models within risk assessment—is fundamental to value‐neutrality. Even in the absence of formal uncertainty and decision analysis, value‐neutral risk assessments offer the most useful and informative kinds of predictions. This is because they offer a sense of consistency in priority setting, and moreover allow publics and decisionmakers to bring *their own* interests, values, and decision rules to bear on discussions about how to act given our best understanding of (future) states of the world. None of this is to say that value judgments be damned, but rather that they should be accounted for within a structured approach to decision analysis or an informed governance processes, rather than embedded within the core inferential tasks of risk assessment in an informal manner.

## Footnotes

1 The Neyman–Pearson paradigm is concerned with the practical consequences of accepting or rejecting a hypothesis. As such, it is more directly relevant for risk assessment, given that such assessments take place within a broader decision‐making context.

2 We can recast this argument in triplet form, where the scenarios in question are acceptance and rejection of the hypothesis (or more precisely, as I shall argue, the specific actions that would follow acceptance or rejection), the probability relates to the chances of erroneously accepting or rejecting the hypothesis, and the consequences are valuations of the outcomes that stem from acceptance or rejection. Of course, at the risk assessment stage, the specific actions may not yet have been identified. This is part of the reason the AfIR is untenable, as I shall argue later.

3 I owe this point to a reviewer.

4 Although proponents of the AfIR do not explicitly preclude formal uncertainty or decision analysis, these methods do not feature much within their writings (e.g., Douglas, 2000, 2009; Rudner, 1953). Crucially, at no point have proponents of the AfIR proposed a formal method for combining judgments of probabilities and consequences to derive inferential thresholds such as *p*‐values (Kaivanto & Steel, 2017). When they have discussed formal methods of uncertainty or decision analysis, it is typically to express skepticism that these methods can be used to circumvent social or ethical judgments (e.g., Havstad & Brown, 2017).

5 I owe this interpretation to a reviewer.

6 Again, I owe these qualifiers to a reviewer.

7 A reviewer emphasized that many model parameters will not have direct physical interpretations, and in such cases it may be unreasonable to expect experts to have any useful intuitions about their respective distribution functions.

8
*De minimis* decision rules, based on the notion that “the law does not concern itself with trifles,” set out risk thresholds that are negligible (Peterson, 2002), whereas *de manifestis* rules set out risk thresholds deemed unacceptably high.

9 “Overregulation presents excess costs to the industries that would bear the costs of regulations. Underregulation presents costs to public health and to other areas affected by damage to public health” (Douglas, 2000). “If we wrongly chose the threshold model, many people would get sick and die prematurely of cancer; the moral cost, in this case, is very high, not to mention the economic costs of treating these individuals. On the other hand, if we wrongly chose the no‐threshold model, the worst that would happen is that corporate profits would be slightly reduced” (Biddle, 2013). “For HTT [high‐throughput toxicology], this would mean taking into account (at least) that some epistemic errors will lead to detrimental effects on human health and the environment (due to underregulation); others will lead to detrimental economic effects (due to overregulation)” (Hicks, 2018). “[Disutilities] associated with outcomes stemming from…not regulating a chemical that is actually toxic (i.e., harms to public health and the environment)….[costs] associated with…regulating a chemical that is non‐toxic (i.e., unnecessarily burdensome and costly regulation)” (Frank, 2017). “If you want to be absolutely sure that you do not say that the chemical is safe when it in fact is not (because you value safety, precaution, welfare of potential third parties), you should decrease your rate of type II errors, and thus increase your statistical significance factor and your rate of type I errors. If you want to avoid ‘crying wolf’ and asserting a link where none exists (because you value economic benefits that come with avoiding overregulation), you should do the reverse” (Brown, 2013).

10 For example, airbags may protect adults but kill children; gas mileage standards may protect the environment at the cost of thousands of lives annually, as they encourage manufacturers to sacrifice sturdiness for fuel efficiency; structural risk mitigation measures may create a “levee effect,” whereby a (false) sense of security encourages populations to settle in floodplains and court catastrophe should the levee overtop; drug lags stemming from stringent testing requirements may protect the public from potential adverse effects of untested pharmaceuticals, while diminishing the health of those who urgently need them; bans on carcinogens in food additives may lead consumers to use noncarcinogenic products, which nevertheless carry even greater health risks, and so on (Busby et al., 2012; Graham & Wiener, 1995; MacGillivray et al., 2011; Sunstein, 1990; Viscusi, 1996).

11 Risk management interventions often have ancillary benefits; compliance costs are routinely overestimated in regulatory proceedings; and many risks are ignored when it appears it is simply too challenging to establish the costs of management options.

12 “Savage carefully limited Bayesian decision theory to ‘small worlds’ in which all alternatives, consequences, and probabilities are known. And he warned that it would be ‘utterly ridiculous’ to apply Bayesian theory outside a well‐defined world—for him, ‘to plan a picnic’ was already outside because the planners cannot know all consequences in advance” (Gigerenzer & Marewski, 2015).
